# Anabolic and Pro-metabolic Functions of CREB-CRTC in Skeletal Muscle: Advantages and Obstacles for Type 2 Diabetes and Cancer Cachexia

**DOI:** 10.3389/fendo.2019.00535

**Published:** 2019-08-02

**Authors:** Rebecca Berdeaux, Chase Hutchins

**Affiliations:** ^1^Department of Integrative Biology and Pharmacology, Center for Metabolic and Degenerative Diseases, The Brown Foundation Institute of Molecular Medicine, McGovern Medical School, University of Texas Health Science Center Houston, Houston, TX, United States; ^2^Graduate Program in Biochemistry and Cell Biology, The MD Anderson-UTHealth Graduate School of Biomedical Sciences, Houston, TX, United States

**Keywords:** CREB, CRTC, SIK, cAMP, muscle hypertrophy, muscle atrophy, type 2 diabetes, cancer cachexia

## Abstract

cAMP is one of the earliest described mediators of hormone action in response to physiologic stress that allows acute stress responses and adaptation in every tissue. The classic role of cAMP signaling in metabolic tissues is to regulate nutrient partitioning. In response to acute stress, such as epinephrine released during strenuous exercise or fasting, intramuscular cAMP liberates glucose from glycogen and fatty acids from triglycerides. In the long-term, activation of Gs-coupled GPCRs stimulates muscle growth (hypertrophy) and metabolic adaptation through multiple pathways that culminate in a net increase of protein synthesis, mitochondrial biogenesis, and improved metabolic efficiency. This review focuses on regulation, function, and transcriptional targets of CREB (cAMP response element binding protein) and CRTCs (CREB regulated transcriptional coactivators) in skeletal muscle and the potential for targeting this pathway to sustain muscle mass and metabolic function in type 2 diabetes and cancer. Although the muscle-autonomous roles of these proteins might render them excellent targets for both conditions, pharmacologic targeting must be approached with caution. Gain of CREB-CRTC function is associated with excess liver glucose output in type 2 diabetes, and growing evidence implicates CREB-CRTC activation in proliferation and invasion of different types of cancer cells. We conclude that deeper investigation to identify skeletal muscle specific regulatory mechanisms that govern CREB-CRTC transcriptional activity is needed to safely take advantage of their potent effects to invigorate skeletal muscle to potentially improve health in people with type 2 diabetes and cancer.

## Introduction

Many chronic human diseases are either caused or accompanied by skeletal muscle dysfunction and loss of muscle mass (1). In obesity and type 2 diabetes, reduced skeletal muscle glucose uptake and inefficient use of glucose and lipid-derived metabolites contributes to whole body metabolic dysfunction (2, 3). Reduced skeletal muscle mass also underlies impaired metabolism in type 2 diabetes (4). Muscle wasting and metabolic dysfunction are even more severe in cancer cachexia (5, 6). Although pharmacologic strategies to reverse these effects through activating key pro-hypertrophic and inhibiting inflammatory signaling pathways are being actively investigated (1), GPCR-activated cAMP signaling is an additional pathway with salutary effects on skeletal muscle metabolism and growth (7, 8). Chronic activation of intracellular cAMP signaling, either through exercise training or chronic administration of agonists to Gs-coupled GPCRs, results in transcriptional activation of genes that reduce muscle protein breakdown and increase mitochondrial biogenesis and metabolism, ultimately promoting muscle growth, and metabolic function [reviewed in (8)]. Conversely, muscle-specific deletion of *Gnas* (which encodes Gαs) in mice causes loss of muscle mass and poor glucose tolerance in concert with reduced mitochondrial abundance and function (9).

**Figure 1:**
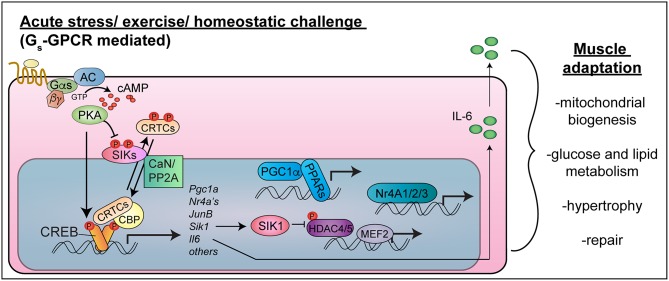
CREB-CRTC function in skeletal muscle. Exercise or acute stress activates Gs-coupled GPCRs to stimulate cAMP-PKA signaling. PKA phosphorylates CREB in the nucleus, leading to CBP recruitment, and phosphorylates and inhibits SIKs, leading to CRTC de-phosphorylation in concert with phosphatase activation (CaN/PP2A). CREB-CRTC-CBP complexes activate target genes encoding transcription factors (PGC1-alpha, Nr4A family nuclear hormone receptors, JunB), signaling mediators (SIK1) and myokines (IL-6), which collectively stimulate mitochondrial biogenesis, improve nutrient uptake and metabolism, stimulate hypertrophy and allow muscle repair. Targeting these pathways in a muscle cell autonomous manner would be expected to have salutary effects on skeletal muscle health and function in type 2 diabetes and cancer cachexia (Note: the nucleus is not drawn to scale).

As in other cell types, the cAMP response element binding protein (CREB) is activated in response to cAMP signaling and other intracellular signaling pathways in myocytes (Figure 1). CREB becomes activated in skeletal muscle after intense exercise (10–12), fasting (13), nerve activity or muscle depolarization (10, 14, 15), and necrotizing injury (16). cAMP signaling simultaneously activates CRTCs (CREB regulated transcriptional coactivators), which interact with CREB to drive transcription of cAMP-regulated genes (17). Because of their implicated functions in muscle hypertrophy and metabolic adaptions to exercise, CREB, CRTCs, and their target genes represent potential therapeutic targets to sustain skeletal muscle mass and function in diabetes and cancer.

This mini-review focuses on the known roles and transcriptional targets of CREB and CRTCs in regulation of skeletal muscle function and adaptation. We highlight how the transcriptional activity of CREB-CRTC regulates muscle function and explore dysregulation of the cAMP-CREB-CRTC pathway in skeletal muscles in the context of type 2 diabetes and cancer cachexia. Finally, we discuss literature showing that although there is potential to harness these endogenous pathways to maintain muscle mass and improve function in both disease states, there are also risks due to diabetogenic and tumor-promoting roles of CREB-CRTCs in non-skeletal muscle organs and tumor cells. Identification of skeletal muscle-specific mechanisms of CREB-CRTC regulation could allow development of novel therapeutic strategies to target this pathway to improve muscle health and avoid off-target effects in individuals with metabolic disease or cancer.

### Literature Search Strategy

The authors conducted independent, unbiased reviews of published literature using numerous combinations of search strategies (e.g., CREB or CRTC and cancer, cAMP cachexia, CRTC metabolism, Gs skeletal muscle, GPCR skeletal muscle) in PubMed and GoogleScholar. We examined citations within those articles and subsequent articles that cited them. We sought association of muscle-expressed Gs-coupled GPCRs with cancer. Articles with contradictory information were not excluded. We focused discussion on articles with mechanistic findings related to skeletal muscle autonomous and non-autonomous functions of CREB-CRTCs, their target genes and upstream activating Gs-coupled GPCRs in skeletal muscle hypertrophy, metabolism, and cancer.

## Regulation of CREB-CRTC Transcription Factors in Skeletal Muscle

cAMP activates CREB through direct PKA-mediated phosphorylation on serine 133, which can also be phosphorylated by other kinases [Figure 1; reviewed in (18)]. Phospho-CREB then recruits the histone acetyltransferase CREB binding protein (CBP). In addition, cAMP signaling leads to activation of CRTC co-activators, also known as TORCs (19, 20). CRTCs are held in latent cytosolic complexes with 14-3-3 proteins due to phosphorylation (21, 22) by AMPK-related kinases: salt inducible kinases (SIK1, SIK2, and SIK3) (21, 23), AMPKα1, AMPKα2 (24), and MARK2 (25). cAMP-PKA signaling inhibits these kinases, and calcium or growth-factor signaling activates calcineurin or protein phosphatase 2A (PP2A) (21, 22, 26). The convergence of these signals results in CRTC de-phosphorylation and nuclear entry. CRTC2 interacts in transcriptional complexes with CREB (19), CBP (27, 28), NONO (29), and KAT2B (30) to stimulate expression of genes containing CREB binding sites (17, 31).

In pancreatic beta cells, CRTC2 functions as a coincidence detector of both depolarization (Ca^2+^ dependent activation of calcineurin phosphatase) and incretin hormones (cAMP-PKA inhibition of SIK kinases), culminating in CRTC2 nuclear entry (21). In cultured skeletal myocytes, CRTCs appear to be regulated in a similar manner: catecholamines and calcium signaling synergize to induce CRTC2 and CRTC3 dephosphorylation and activation (32). The CRTC phosphatase in skeletal muscle has not been determined. All of the known CRTC kinases are expressed in skeletal muscle, either at baseline (33, 34) or in response to stimulus (35), so it will be interesting to examine CRTC activity in muscles of mice lacking these kinases. Based on similarity to hepatocytes, in which CRTC phosphorylation remained largely intact with single genetic knockout of *Sik1* (36) or *Sik2* (37) or with triple knockout of *Sik2*/*Ampk-a1*/ *Ampk-a2* (37), there might be functional redundancy among CRTC kinases in skeletal muscle.

## CREB-CRTC Functions and Target Genes in Skeletal Muscle Adaptation and Dysfunction

### Skeletal Muscle Phenotypes in Mice With Altered CREB-CRTC Function

In adult skeletal muscle, exercise and other catabolic conditions activate CREB, which is associated with expression of genes involved in metabolism [(10–12); and Section CREB-CRTC Target Genes in Skeletal Muscle]. *Creb* knockout in mice causes perinatal lethality (38) accompanied by defective muscle progenitor cell proliferation (39) or no phenotype due to developmental compensation by related proteins (40). Transgenic over-expression of dominant-negative CREB (A-CREB) in postnatal skeletal muscles of mice caused muscle degeneration that was associated with reduced expression of MEF2 target genes and was rescued by reintroduction of SIK1 (34). Unfortunately, the developmental and degenerative phenotypes preclude the use of these animal models to study CREB function in muscle hypertrophy or metabolism in adult animals. Surprisingly, no studies to date have utilized muscle-specific Cre recombinase drivers with available conditional knock-out or knock-in alleles to achieve muscle-specific *Creb* knockout (41) or inducible A-CREB expression in postnatal muscles (42). Knock-in of a mild CREB gain-of-function mutant [CREB(Y134F)] did not cause hypertrophy or patent metabolic alterations, but did increase myoblast proliferation (16).

All three CRTC proteins are expressed in cultured skeletal myocytes (32, 43). CRTC2 and CRTC3 proteins are also expressed in mouse skeletal muscle, where they are activated (dephosphorylated) in response to intense exercise (32). Unlike *Creb* knockout mice, *Crtc1, Crtc2*, and *Crtc3* single knockout mice develop normally without reported muscle defects (44–47). Mice lacking *Crtc3* in all tissues (47) or selectively in brown fat and skeletal muscle (48) have reduced adiposity and increased brown adipose thermogenesis (47, 48). Activation of CRTC3 in adipose would therefore be expected to worsen metabolism in people with type 2 diabetes. Indeed, activating *CRTC3* mutations are associated with increased adiposity in humans (47).

In skeletal muscle, CRTCs also regulate metabolism. Forced expression of CRTCs in primary myotubes promoted mitochondrial biogenesis and improved mitochondrial oxidative capacity via transactivation of *Ppargc1a* (encoding PGC-1α, the master regulator of mitochondrial biogenesis) (49). Bruno et al., established *in vivo* roles for CRTCs in skeletal muscle metabolism by demonstrating that transgenic mice with doxycycline inducible expression of CRTC2 exhibit myofiber hypertrophy, increased intramuscular glycogen and triglycerides, and enhanced exercise performance (32). Muscle over-expression of CRTC2 or CRTC3 also prevented protein degradation, which was associated with activation of the SIK1-class II HDAC pathway by transcriptional induction of *Sik1* (34, 50) as well as with increased IGF1-AKT signaling due to transcriptional activation of *Ppargc1a4*, encoding PGC-1α4 (51). The authors suggest that CRTCs mediate transcriptional reprogramming in skeletal muscle after strenuous exercise by activation of transcriptional targets (section CREB-CRTC Target Genes in Skeletal Muscle) known to drive mitochondrial biogenesis, myofiber hypertrophy, and energy storage (32). It would be important to test whether over-expression of CRTC2 or CRTC3 in skeletal muscle *in vivo* is capable of stimulating mitochondrial gene expression and oxidative capacity, as in primary skeletal muscle cells *in vitro* (49).

### CREB-CRTC Target Genes in Skeletal Muscle

#### PGC-1alpha

CREB-CRTC complexes bind directly to the gene encoding PGC-1α (32, 52), which is well-known to drive mitochondrial biogenesis in skeletal muscle (53). Forced expression of CRTCs stimulated *Pgc-1alpha* mRNA levels in skeletal myocytes (32, 49). Interestingly, CRTCs and CREB preferentially associated with the distal region of the *Pgc-1alpha* gene in myocytes (32), which activates expression of the pro-hypertrophic *Pgc1-alpha4* isoform (51). Isoproterenol had a much stronger effect on CRTC3 occupancy in the same genomic region than on CRTC2, indicating that the signaling controlling these two isoforms may diverge in skeletal muscle cells. Notably, CRTC3, but not CRTC2, is dephosphorylated by PP2A after MAP kinase dependent priming in a non-muscle cell line (26). It is therefore possible that CRTC3 is more sensitive to isoproterenol than CRTC2 due to simultaneous activation of Gαs-activated cAMP signaling (inhibit SIKs) and Gβγ-activated MAP kinase signaling (54) (enhance PP2A recruitment). Increased expression of both *Pgc-1alpha* isoforms in skeletal muscle would be expected to improve both muscle metabolism and strength in type 2 diabetes and cancer cachexia.

#### NR4A Nuclear Hormone Receptors

*Nr4a* family nuclear hormone receptors are direct CREB target genes (55–58) that are strongly induced in response to exercise (in mice, rats, and humans), β-adrenergic receptor activation, cAMP signaling, or overexpression of CRTCs in muscle cells (16, 32, 34, 59–63). Transgenic mice with overexpression or knockout of *Nr4a1, Nr4a2*, or *Nr4a3* have revealed that NR4A receptors have numerous beneficial effects on skeletal muscle metabolism and growth (59). For example, NR4A1/Nurr77 supports myogenesis (64), muscle fiber hypertrophy (65), insulin sensitivity, glucose uptake (66, 67), and lipid metabolism (68). In mice fed high fat diet, MED13 represses *Nr4a2/Nurr1* transcription, which normally directly induces transcription of *Glut4* and additional genes to promote glucose uptake and utilization (69). NR4A3/NOR-1 stimulates oxidative metabolism in skeletal muscle. siRNA-mediated knockdown of *Nor1* (also called Nr4a3) in skeletal myocytes reduced palmitate oxidation (70). Conversely, transgenic expression of NOR-1 in mouse muscles caused a lean phenotype and increased exercise endurance, with increased muscle mitochondrial density and autophagy. At the molecular level, these phenotypes were associated with increased expression of *myoglobin* and genes involved in glycogen storage and aerobic metabolism (60, 71). Muscle phenotypes have not been reported in viable NOR-1 knockout mice (72).

#### IL-6

Interleukin-6 (IL-6) is a pro-inflammatory cytokine and myokine secreted from many cell types including skeletal muscle, in which IL-6 has pleiotropic functions (73). Basal or transient IL-6 signaling promotes muscle regeneration and growth in response to muscle damage, but chronically high IL-6 signaling in hyper-inflammatory states such as type 2 diabetes and cancer promotes muscle atrophy through activation of the JAK-STAT pathway in conjunction with elevated TNF-alpha signaling (74, 75). The *Il-6* gene has a full CREB binding site (58). In C2C12 myotubes, CREB sustains basal *Il6* expression (76) and cooperates with NF-κB in transcriptional activation of the *Il6* promoter in myocytes treated simultaneously with a beta-adrenergic agonist and TNF-alpha (77). Muscle *Il6* expression also increases after exercise (62, 76), but it has not been determined whether CREB-CRTCs regulate *Il6* in this context.

#### JunB

A member of the AP-1 family of transcription factors, JunB is an immediate early gene induced by CREB (78). In human skeletal muscle, *JUNB* expression is induced by exercise (79, 80) or physiologic hyperinsulinemia (81). In mice, JunB is pro-hypertrophic: dexamethasone-induced atrophy in C2C12 myotubes dramatically reduces *JunB* expression, and JunB knock-down reduces muscle mass *in vivo*, whereas over-expression stimulates muscle hypertrophy by counteracting FoxO3 binding to atrogene promoters (82). Although *JunB* expression in skeletal muscle has not been directly linked to CREB activity, it is possible that JunB is part of an anabolic program induced by CREB-CRTC2 during exercise.

#### SIK1

Salt inducible kinase 1 (*Sik1*) is a direct cAMP-regulated CREB target gene in myocytes and most other cells types (34, 83–85). SIK1 phosphorylates not only CRTCs but also class II histone deacetylases (HDACs), thereby indirectly activating MEF2 (34, 86–88). In transgenic mice expressing dominant-negative A-CREB in skeletal muscle, SIK1 re-expression rescues muscle degeneration and restores class II HDAC phosphorylation (34). In cultured myocytes, knock-down of *Sik1* mRNA impairs differentiation *in vitro* (35). Muscle deletion of the *Sik1* catalytic domain in mice leads to improved muscle glucose uptake after high fat diet feeding without developmental defects, indicating that SIK1 regulates muscle metabolism (36). It will be interesting in future studies to determine how SIK1 regulates muscle metabolism and whether it is a key CREB-CRTC effector in metabolic adaptation to exercise.

### Potential Therapeutic Value and Complications of CREB-CRTC Activation in Type 2 Diabetes and Cancer Cachexia

From the above discussion, it is clear that cAMP signaling to CREB-CRTCs in skeletal muscle drives gene expression programs capable of increasing muscle growth, metabolic efficiency, and exercise performance. Each of these functions would be beneficial to improve metabolism and overall health in patients with type 2 diabetes and cachexia. This might imply that pharmacologic activation of Gs-coupled GPCRs in skeletal muscle or pharmacologic blockade of CRTC inhibitory kinases would have salutary effects on skeletal muscle in people with these diseases. However, recent reports established that activation of Gs-signaling as well as CREB-CRTCs in other organs and in tumor cells would be expected to worsen metabolic disease and cancer. Therefore, to harness this pathway for therapeutic benefit, it will be important to identify CREB-CRTC regulatory mechanisms specific to skeletal muscle.

#### GPCR Signaling

Several Gs-coupled GPCRs expressed in skeletal muscle have been documented to promote hypertrophy and to improve muscle metabolism (Table 1). β_2_-adrenergic agonists, such as clenbuterol and formoterol, have shown great promise in maintaining skeletal muscle mass in animal models, but have undesirable cardiovascular side effects [reviewed in (7, 108, 109)] that might be avoided by newer-generation agonists with functional selectivity for skeletal muscle (110). Similarly, stimulation of CRFR2 with chronic administration of urocortin 2 (94–97) or transgenic expression of urocortin 3 (111) promotes muscle hypertrophy. However, CRFR2 signaling can either blunt (93) or augment (111) insulin action in skeletal muscle, depending on the ligand. Thus, stimulating cAMP signaling selectively in skeletal muscle, but not liver or heart, through β_2_-adrenergic receptors, Frizzled-7 (101), TGR-5 (103) or urocortin3-CRFR2 signaling (111), might be useful for treatment of type 2 diabetes.

**Table 1:** Gs-coupled GPCRs in skeletal muscle and tumors.

**Receptor**	**Roles in skeletal muscle hypertrophy/metabolism**	**Association with cancer**
β-Adrenergic receptors (β_1_, β_2_, β_3_)	Glycogen breakdown, transcription, excitation-contraction coupling metabolic adaption, hypertrophy (7)	β_1_ and β_3:_ Over-expressed in breast cancer (89); contributes to malignancy in breast (89), prostate (90), lung (91), and many others (92)
CRFR2	Inhibit insulin sensitivity (93) Reduce Atrophy (94–97)	Inhibits vascularization and growth of several cancer cell types; loss is associated with aggressive renal cancer (98–100)
Frz7 (Frizzled7)	Hypertrophy (101)	Upregulated in hepatocellular carcinoma, breast cancer, wilms tumor, gastric cancer, ovarian cancer, melanoma (102)
TGR5	Hypertrophy (103)	Overexpressed (gastric and esophageal adenocarcinomas); associated with poor prognosis; promotes growth and migration in small cell lung cancer cells (104–106); protective against renal cancer cell proliferation (107)

*Table indicates muscle phenotypes induced by the indicated GPCRs and the association of the GPCRs with cancer*.

In sharp contrast, stimulating Gs-coupled GPCRs in cases of cachexia would be expected to have deleterious effects on malignancy. Activating mutations of the cAMP-producing G protein Gαs (*Gnas*) are observed in ~5% of all human tumors (112) and 40–75% of pancreatic cancers (113). The literature points to a trend of cancer-promoting effects of many of the G_S_-coupled GPCRs that drive hypertrophy in skeletal muscle (Table 1). For example, β_1_ and β_3_ adrenoreceptors are overexpressed in breast tumors, when compared to normal breast tissue, and non-selective beta-blockers reduced proliferation indices in early stage breast tumors (89). Similar observations were made in many other cancers, either through *in vitro* studies or analysis of human specimens and correlation of clinical outcome with administration of beta-blockers (Table 1). The same is true of Fzd7 and TGR5, which are generally associated with worse cancer phenotype (proliferation, invasion, and metastasis) [(102, 104) and Table 1]. Thus, pharmacological activation of several of the Gs-coupled GPCRs that are expressed in skeletal muscle would be a poor choice for therapeutic targeting of muscle loss in cancer cachexia. The notable exception is CRFR2, which has pro-hypertrophic effects in skeletal muscle but tumor suppressive effects via inhibition of vascularization and tumor cell proliferation (98–100).

#### CREB-CRTCs and SIKs

Activation of CRTC2 or CRTC3 in muscle or myotubes by over-expression has beneficial effects on myocyte size (32) and metabolism (49), rendering these proteins potentially promising candidates for sustenance of muscle mass and function. It would be important to test whether transgenic expression of CRTCs in skeletal muscle could overcome atrophy or improve muscle metabolism in mouse models of cancer cachexia or type 2 diabetes. One novel therapeutic approach could be the use of bioavailable SIK inhibitors to potentiate nuclear CRTC entry (85). Currently available SIK inhibitors target all SIKs, and the most bioavailable inhibitor YKL-05-093 activates CRTC2 and class II HDACs in mice and stimulates bone formation (114). YKL-05-093 had no reported effects in lean animals, but SIK inhibitors might be expected to cause complex metabolic changes in obese animals due to functions of SIKs in liver, adipose tissue, and skeletal muscle (36, 37, 83, 115–118). More research is needed on regulation and functions of CRTCs and SIKs in skeletal muscle to predict the therapeutic value of targeting this pathway for type 2 diabetes.

Similar to GPCRs, strategies to pharmacologically activate CREB-CRTCs for cancer cachexia is questionable because gain of CREB-CRTC function in tumor cells is strongly associated with proliferation, survival, metabolic adaptation, and invasion [comprehensively reviewed in (119)]. The SIKs have emerged as tumor regulators as well. SIK1 is downregulated in several cancer types (120, 121) and has been shown to be a tumor suppressor by activating anoikis (anchorage-dependent cell death) to inhibit metastasis (120) by suppressing metabolic reprogramming (122) and epithelial to mesenchymal transitions (123). On the other hand, SIK2 activity at the centrosome is required for mitotic spindle assembly and survival of ovarian cancer cells (124, 125). A SIK2 inhibitor (ARN-3236) synergizes with paclitaxel to improve outcome in preclinical ovarian cancer models (126). The effects of SIK inhibition on muscle mass have not been evaluated, but due to the pro-oncogenic functions of CRTCs in tumor cells, strategies to activate CRTCs to increase muscle mass in cancer cachexia would require identification of skeletal muscle-specific mechanisms of CRTC regulation.

## Concluding Remarks

Long known for its role as a second messenger coordinating skeletal muscle nutrient utilization with functional demands, cAMP has also been appreciated for its ability to stimulate hypertrophic growth and long-term metabolic adaptation. CREB-CRTCs are key cAMP effectors that contribute to muscle adaptation through activation of transcriptional programs that increase hypertrophic growth, mitochondrial biogenesis, metabolic efficiency, and muscle performance. Therefore, targeting CREB-CRTCs could be highly advantageous to sustain muscle mass and improve muscle function in people with type 2 diabetes and cancer cachexia. However, functions of CREB-CRTCs in other metabolic tissues as well as in tumor cells present significant obstacles to developing therapeutic approaches to safely target this pathway. Targeting CRTCs has the most potential for improving metabolism in type 2 diabetes, but should be approached with caution in cancer cachexia due to oncogenic effects of these proteins. Identification of specific signaling mechanisms that regulate CREB-CRTCs in muscle cells could enable the field to harness the potential of these proteins to improve skeletal muscle health.

## Author Contributions

RB developed the thesis, designed the manuscript organization, and prepared the figure. RB and CH prepared the table, and wrote and edited the manuscript. Both authors approved the final version.

### Conflict of Interest Statement

The authors declare that the research was conducted in the absence of any commercial or financial relationships that could be construed as a potential conflict of interest.

## Abbreviations

A-CREB: acidic-CREB (dominant-negative CREB)
AKT: Protein Kinase B
AMPK: adenosine monophosphate activated protein kinase
cAMP: cyclic adenosine monophosphate
CBP: CREB binding protein
CREB: cAMP response element binding protein
CRFR: corticotrophin-releasing hormone receptor
CRTC: CREB regulated transcriptional coactivator
FoxO3: forkhead box protein
GPCR: G Protein-coupled receptor
HDAC: histone deacetylate
IGF-1: insulin like growth factor 1
IL-6: interleukin 6
JAK: Janus Kinase
MAPK: mitogen activated protein kinase
MED13: mediator complex subunit 13
MEF2: myocyte enhancer factor-2
NF-κB: nuclear factor kappa B
NR4A: Nuclear receptor family 4A
PGC-1α: Peroxisome proliferator-activated receptor gamma coactivator 1-alpha
PKA: Protein Kinase A
PP2A: protein phosphatase 2A
SIK: salt inducible kinase
STAT: Signal transducer and activator of transcription proteins
TNF-alpha: tumor necrosis factor alpha
TORC: transducer of regulated CREB activity.
